# Risk Taking Runners Slow More in the Marathon

**DOI:** 10.3389/fpsyg.2019.00333

**Published:** 2019-02-27

**Authors:** Robert O. Deaner, Vittorio Addona, Brian Hanley

**Affiliations:** ^1^Department of Psychology, Grand Valley State University, Allendale, MI, United States; ^2^Department of Mathematics, Statistics, and Computer Science, Macalester College, Saint Paul, MN, United States; ^3^Carnegie School of Sport, Leeds Beckett University, Leeds, United Kingdom

**Keywords:** DOSPERT, decision making, discomfort, distance running, pacing, risk taking, road racing, training

## Abstract

Much research has explored the physiological, energetic, environmental, and psychological factors that influence pacing in endurance events. Although this research has generally neglected the role of psychological variation across individuals, recent studies have hinted at its importance. Here we conducted an online survey of over 1,300 marathon runners, testing whether any of five psychological constructs – competitiveness, goal achievement, risk taking in pace (RTP), domain-specific risk taking, and willingness to suffer in the marathon – predicted slowing in runners’ most recent marathons. Analyses revealed that RTP – the extent to which runners agreed that they began the marathon at a pace that was so fast that it would jeopardize their capacity to maintain this pace throughout the event – was a robust predictor of marathon slowing. RTP proved a substantial predictor even in regression models controlling for the other psychological constructs, training, experience, and other known pacing correlates. This result suggests that marathoners consider trade-offs when making pacing decisions, and that individuals vary in their pacing decision making.

## Introduction

Successful completion of endurance events, such as long distance running, biking, and swimming, requires that individuals effectively allocate their energetic resources throughout the event. This allocation, and the associated changes in speed throughout the event, is referred to as pacing (Foster et al., 2004). Pacing has long been of interest to scientists, and they have characterized successful pacing trajectories and identified physiological, psychological, energetic, and environmental factors that influence pacing (Abbiss and Laursen, 2008; Tucker and Noakes, 2009; Roelands et al., 2013).

Pacing research has traditionally employed small sample sizes and focused on elite or near-elite competitors, such as those participating in Olympic and World Championship marathons (Hanley, 2016) or those setting marathon world records (Diaz et al., 2018). Several recent studies, however, have explored pacing in large samples of non-elite marathon runners (Santos-Lozano et al., 2014; Trubee et al., 2014; Deaner et al., 2015a; Hubble and Zhao, 2016; Allen et al., 2017; Krawczyk and Wilamowski, 2017; Nikolaidis and Knechtle, 2017; Smyth, 2018). These non-elites are of interest because they vary greatly in their ability, motivation, and experience (Masters et al., 1993; Ogles and Masters, 2003), as well as their pacing. This variation should allow scientists to identify sources of pacing variation across individuals, and some evidence has already emerged that psychology may be a crucial factor. Specifically, two studies found that runners who show greater slowing in the marathon generally exhibit a larger discrepancy between their pre-race self-forecast and their actual performance; this correlation may arise because overly confident runners tend to start the marathon too fast, which leads to greater slowing compared to runners with more reasonable expectations and less aggressive starting paces (Hubble and Zhao, 2016; Krawczyk and Wilamowski, 2017).

In the current study, we further explore the role of psychology in explaining pacing variation across marathon runners. We do this by analyzing over 1,300 runners’ self-reports regarding their most recent marathon, particularly their finishing time, pacing, training, running experience, and psychology. Our first objective is to test whether any of the following five psychological constructs are associated with pacing: competitiveness, (motivation for) goal achievement, risk taking in pace (RTP), domain-specific risk taking (DOSPERT), and willingness to suffer in the marathon (WSM).

Greater competitiveness and goal achievement have been consistently linked with faster marathon finishing times (Masters et al., 1993; Ogles and Masters, 2003), and faster finishing times are associated with more even pacing (Haney and Mercer, 2011; March et al., 2011; Renfree and Gibson, 2013; Santos-Lozano et al., 2014; Trubee et al., 2014; Deaner et al., 2015a; Hubble and Zhao, 2016; Krawczyk and Wilamowski, 2017; Nikolaidis and Knechtle, 2017; Smyth, 2018). We thus predict that greater competitiveness and goal achievement will be associated with more even pacing.

Decision making is an important aspect of optimizing marathon performance, as runners must continually choose how and when to invest their energy throughout the race (Smits et al., 2014). For example, before a race begins, the runner might decide to start with a conservative pace (given their ability) that makes it likely they will achieve a satisfactory performance (Smits et al., 2014) or to start relatively aggressively in the hope of maintaining that pace and achieving an exceptional performance. Trade-offs are inevitable: the more aggressive the early pace, the less likely the runner is to achieve their goal(s) (Markle et al., 2018). Thus, the construct of RTP addresses the extent to which a runner begins the race at a fast pace (given their ability) that could lead to an excellent performance but which puts them at risk of slowing dramatically later in the race. We predict that greater RTP should be associated with greater slowing (i.e., less even pacing).

Many scientists hold that risk taking is domain-specific, rather than domain-general (Blais and Weber, 2006; Figner and Weber, 2011; Fox and Tannenbaum, 2011). In this view, for instance, a person who is extremely averse to take risks regarding their health might be quite likely to gamble on a sporting event or to begin a marathon with an aggressive pace. However, considerable evidence supports the hypothesis that risk taking is partly domain-general (Frey et al., 2017; Highhouse et al., 2017). If the domain-general view is correct, then scores from an instrument that assesses risk taking across domains, the DOSPERT scale (Blais and Weber, 2006), should be associated with greater slowing. Although the DOSPERT scale was developed to assess several supposedly independent risk taking domains, the total score can be considered a domain-general risk taking measure (Highhouse et al., 2017).

The construct we call WSM addresses the extent to which a runner is willing to experience discomfort during their marathon. From a physiological point of view, the body produces feelings of discomfort to inform the brain that the body is being pushed to its limits (i.e., running relatively fast) and that negative consequences (e.g., passing out; long-term damage to muscles or organs) could occur if the workload is not decreased (Tucker and Noakes, 2009). Runners typically experience substantial discomfort toward the end of the marathon, particularly if they are slowing substantially (e.g., “hitting the wall”) (Buman et al., 2008), and runners are generally aware of this possibility (Smits et al., 2014). Given this possibility and the trade-offs discussed in the RTP paragraph above, we predict that greater WSM will be associated with greater slowing.

Our second objective is to test whether any associations we discover between these five psychological constructs and pacing remain once we control for potential confounding variables. As noted above, it is already established that faster finishing times are associated with more even pacing, so finishing time will likely be an important variable to control. In addition, more even marathon pacing is known to be associated with having greater experience (Deaner et al., 2015a), being older (March et al., 2011; Trubee et al., 2014; Deaner et al., 2015a; Krawczyk and Wilamowski, 2017; Nikolaidis and Knechtle, 2017), and being female (March et al., 2011; Santos-Lozano et al., 2014; Trubee et al., 2014; Deaner et al., 2015a; Hubble and Zhao, 2016; Krawczyk and Wilamowski, 2017; Smyth, 2018). Thus, these variables will require investigation. Another potential confound is training, including the volume or distance run and frequency and speed of training. Although training has not been previously linked to pacing, training variables have been shown to correlate with finishing times (Tanda and Knechtle, 2015; Gordon et al., 2017), a known correlate of pacing (see references above). Moreover, scientists (Hanley, 2016) and training manuals (Martin and Coe, 1997) agree that proper training is crucial for maintaining an even, or near even, pace in the marathon.

## Materials and Methods

### Research Approval

All human subjects were treated in accordance with established ethical standards. The Chair of the Human Research Review Committee at Grand Valley State University reviewed the study protocol (870392-1) and certified it as approved and exempt from full committee review on March 3, 2016.

### Recruitment

We recruited marathon runners by advertising our study online, in places such as running forums and social media platforms. It also involved researchers RD and BH directly contacting marathoners they knew (including via social media) and inviting them to participate and share the invitation with other marathoners and running clubs. Recruitment began on April 19, 2016. A major increase in recruitment occurred on November 10, 2016 when, at our request, a popular running magazine, Runner’s World, posted a brief online article about our study and invited its readers to participate.

From April 19, 2016 to November 10, 2016, before the Runners’ World article, 324 individuals opened the survey link and consented to participate. After 10 November, 2894 individuals opened the survey link and consented to participate. Approximately 90% of those who participated after November 10 did so before November 17, suggesting that most of our participants learned of our study because of the Runner’s World article. Only responses completed through December 8, 2016 were included in the final data set.

The advertising and consent forms for our study explained that we were conducting a study of pacing in the marathon and that we planned to enroll a large number of marathoners of widely ranging abilities and ages. Marathoners were invited to complete an online survey that would consist of questions addressing the individual’s training, performance, motivation, and pacing in their most recently completed marathon. The survey was expected to take roughly 8–20 min. There was no compensation offered for participating. However, we invited participants to email us if they would like to be added to a list so that we could email them a link to our study once it was published. Only individuals who indicated that they were at least 21 years of age and had completed at least one marathon were eligible to participate.

### Participants

A total of 3,218 individuals opened the survey link and consented to participate, and 51.9% percent of respondents identified themselves as female. The vast majority of respondents were from the United States (78.5%), the United Kingdom (7.0%), or Canada (4.1%), with the remaining respondents representing a wide array of nationalities.

Fifty-one (51) percent of participants completed at least 90% of the survey (*n* = 1,640), with 84.6% providing their sex (*n* = 2,723), 82.9% providing their age (*n* = 2,668), and 41.7% providing sufficient information to calculate our pacing measure (*n* = 1,342). Of the participants who completed over 90% of the survey, 49.1% identified themselves as female. Finally, we had sufficient information to calculate a value for each of the five individual psychological constructs (competitiveness, goal achievement, RTP, DOSPERT, and WSM) for approximately 48–49% of participants, and we had sufficient information to calculate all five constructs for 46.0% of participants (*n* = 1,479). Among participants who provided complete information on psychological items, 49.8% identified themselves as female.

Males in the sample had a mean age of 41.5 years (*SD* = 10.71) and reported finishing their last marathon in 3 h 44.8 min (*SD* = 43.40 min) on average. Females had a mean age of 37.2 years (*SD* = 9.81) and reported finishing their last marathon in 4 h 21.5 min (*SD* = 51.09 min) on average. According to participants’ self-reports of first and second half split times, males slowed by an average of 9.32% in the second half of their last marathon (*SD* = 12.76), whereas females slowed by an average of 10.25% (*SD* = 11.67).

The most common marathons reported in our sample are shown in Table 1 (for those runners that had complete pacing information), along with the percentage of female respondents, the mean slowing value (%), and the mean finishing time (in minutes), which was adjusted for females (see below).

**Table 1:** Most common marathons (among those with complete pacing information), percentage of female respondents, mean slowing (%), and mean adjusted finishing time (in minutes).

Marathon	Respondents	Percent female	Mean slowing (SD)	Mean adjusted finish time (SD)
Detroit	11	81.8	11.68 (11.53)	254.45 (45.21)
Frankfurt	11	18.2	3.28 (5.50)	191.79 (29.36)
Montreal	11	45.5	4.81 (8.91)	224.25 (26.93)
Toronto	11	18.2	3.42 (4.62)	193.60 (19.09)
Amsterdam	12	33.3	2.13 (6.20)	221.19 (26.71)
St. George (UT)	12	58.3	3.28 (6.20)	205.24 (33.68)
Erie (PA)	13	46.2	7.13 (9.20)	209.83 (26.81)
Richmond (VA)	13	61.5	6.22 (7.98)	219.08 (31.05)
Grand Rapids (MI)	14	28.6	5.39 (7.08)	204.46 (28.79)
Columbus (OH)	16	56.3	11.17 (11.60)	236.70 (53.30)
Dublin (IRL)	16	40.0	6.47 (8.40)	225.15 (30.48)
Berlin	17	35.3	3.41 (5.83)	201.38 (40.40)
Philadelphia	17	47.1	8.89 (20.10)	229.76 (56.39)
Twin Cities (MN)	32	50.0	7.59 (8.30)	227.73 (41.33)
Indianapolis	34	61.8	6.72 (7.99)	213.73 (35.35)
London	35	37.1	10.76 (12.05)	201.49 (40.76)
Marine Corps (DC)	70	58.6	15.20 (12.15)	255.92 (47.51)
Boston	78	55.1	11.22 (11.06)	200.08 (23.73)
Chicago	139	51.4	9.63 (11.61)	225.58 (42.75)
New York	178	55.6	8.83 (9.28)	234.01 (43.63)
Other	589	40.8	10.62 (13.96)	226.26 (41.90)
Total	1,329	46.6	9.79 (12.28)	225.12 (42.60)


*Female finishing times were adjusted by dividing them by 1.12 (see section “Analysis”).*

### Representativeness of Sample

Because our sample was self-selected, we attempted to assess its representativeness by comparing it to a cohort of runners who completed similar marathons but did not volunteer to complete our survey. As a source of non-volunteer marathon data, we used the data used in the analysis performed by Deaner et al. (2015a). Deaner et al. (2015a) included data from 14 large (minimum 1,000 finishers) marathons held in the United States in 2011 where halfway and full race data were publicly available. We compared our current sample with that from Deaner et al. (2015a) in terms of its sex and age distributions, the quality of the runners (as measured by marathon finishing time), and their slowing in the second half of a marathon.

Our sample contained information on a higher proportion of females (51.9% vs. 41.5%; effect size = 0.21), and our participants tended to be slightly older than in the Deaner et al. (2015a) data: males were roughly 2.5 years older (41.5 vs. 38.9 years old; effect size = 0.24) and females were approximately 1.7 years older (37.2 vs. 35.5 years old; effect size = 0.17). Moreover, our survey participants reported faster marathon finishing times: males in our data reported finishing, on average, 43 min quicker (3 h 45 min vs. 4 h 28 min; effect size = 0.89), and females reported finishing 33 min quicker (4 h 21 min vs. 4 h 54 min; effect size = 0.64) than males and females, respectively, in the Deaner et al. (2015a) dataset. With regards to pacing, our data suggests less slowing, in particular for males: females in our data reported 1.5% points less slowing (10.2 vs. 11.7; effect size = 0.28), and males reported 6.3% points less slowing, than females and males (9.3 vs. 15.6; effect size = 0.89), respectively, in the Deaner et al. (2015a) data.

### Survey

The survey was presented on Qualtrics, a commercial survey platform. The complete survey can be found in the Supplementary Material. After indicating their consent and eligibility to participate, participants were asked questions about their age, sex, country of residence, personal bests in the marathon and half marathon distance, years of training and/or competing in distance running, and the number of marathons they had started and finished.

Participants were next asked: “What was the most recent marathon you finished (e.g., Boston, London) where you intended to perform as well as you could? For example, if you completed a marathon only as a training run or only to pace a friend and not to run as fast as possible, please disregard that marathon and consider the marathon you finished before that one.” Participants were next asked questions regarding their training for this marathon. The first question was “Did you ever deliberately run at your target pace in training as practice for this marathon?” Options were “regularly,” “a few times,” and “never.” The next question was “Did you ever deliberately run substantially faster than your target pace in training as practice for this marathon? This might be tempo training, threshold training, intervals, fartlek, 10K pace training, or any other training substantially faster than your target marathon pace.” Options were, again, “regularly,” “a few times,” and “never.” The next question was “What was your typical training distance covered each week during preparation for this marathon (not including any tapering period)?” Options were “0–9 miles per week,” “10–19 miles per week,” “20–29 miles per week,”… “120+ miles per week”; there were similar options available for reporting kilometers rather than miles, and these responses were adjusted to miles prior to analysis. The next question was “About how many weeks did you train for this marathon, not including any tapering period?” Options were “4 weeks or fewer,” “5–8 weeks,” “9–12 weeks,” “13–16 weeks,” and “17 weeks or more.” The next question was “About how many training runs or preparation races did you do in preparation for this marathon that were at least 18 miles (29 km) long?” Options were “0 runs/races of at least this length,” “1–2 runs/races of at least this length,” “3–5…,” “6–8…,” and “9 or more…”

Participants were next asked their finishing time and their expected finishing time for this most recent marathon. They were then asked questions about their pacing, including their time to complete the first half of this marathon and (if recalled) their time to complete shorter segments of this marathon including each mile and 5-km segment. Because most participants reported their first half completion time, but not their other segment completion times, we defined pacing using only first and second half completion times (see below for definition of pacing). Participants were then asked further questions about their pacing in this marathon (e.g., pacing relative to pre-race expectations, reasons for slowing, nature of their pacing plan), although these questions were not analyzed for the present study.

Next, participants completed items addressing five psychological constructs that were expected to correlate with pacing. The items representing the constructs were presents in the following order: RTP, WSM, competitiveness, goal achievement, and DOSPERT.

We defined RTP for the first time in this study, and we developed a preliminary measurement scale based on our experience as athletes, coaches, and researchers. The scale was not pre-tested or evaluated by other researchers prior to data collection. RTP was operationalized as the sum of responses to the following five items, all concerning their most recent marathon, each rated on a 5-point Likert scale, ranging from strongly disagree to strongly agree: (1) “For me, achieving a satisfying performance meant running fairly aggressively early in the race”; (2) “I was willing to be pretty conservative with my pace at the beginning so that I could be nearly certain that I’d finish strong” (reverse scored); (3) “I’d hate to finish and realize that I could have gotten a faster time if I had begun the race at a faster pace”; (4) “I started at a pace that was slow enough that I expected to speed up considerably in the second half of the race” (reverse scored); and (5) “I realized that my starting pace was fast enough that I might not be able to maintain it.” From the 1,571 participants who completed all RTP items, we calculated a Cronbach’s alpha of 0.662. Cronbach’s alpha is a scale reliability measure of the internal consistency or agreement among several items purporting to measure the same underlying construct. Because a Cronbach’s alpha below 0.70 is often considered unacceptable (Tavakol and Dennick, 2011), we repeated all analyses involving RTP after removing item #3 so that Cronbach’s alpha was 0.695 for the remaining four items. These results are presented in the Supplementary Material, and they are substantially the same as the results presented below.

We defined WSM for the first time in this study, and we again developed a preliminary measurement scale based on our experiences. The scale was not pre-tested or evaluated by other researchers prior to data collection. WSM was operationalized as the sum of responses to eight items, each rated on a 5-point Likert scale. Each item consisted of a statement and participants indicated how important it was as a goal in their most recent marathon; responses varied from “not a goal,” which had a score of 1, to “a very important goal,” which had a score of 5. The items were: (1) “Discovering how much discomfort I could endure”; (2) “Making sure I finished feeling good” (reverse scored); (3) “Pushing myself to my limits”; (4) “Feeling comfortable during the race” (reverse scored); (5) “Staying healthy” (reverse scored); (6) “Testing my mental toughness”; (7) “Doing everything I could to achieve my fastest possible time”; and (8) “Avoiding physical discomfort” (reverse scored). From the 1,578 participants who completed all WSM items, we calculated a Cronbach’s alpha of 0.663. We repeated all analyses involving WSM after removing items #1 and #6 so that Cronbach’s alpha was 0.718 for the remaining items. These results are presented in the Supplementary Material, and they are substantially the same as the results presented below.

We assessed competitiveness, and goal achievement using the Motivations of Marathoners Scales (MOMS), which consists of 56 items that are rated on a 7-point Likert scale regarding the degree to which the runner considers them a reason for training and running in a marathon or distance race (Masters et al., 1993). The items represent nine internally consistent motivational constructs: affiliation (6 items), competitiveness (4), health orientation (6), life meaning (7), personal goal achievement (6), psychological coping (9), recognition (6), self-esteem (8), and weight concern (4). Each item is rated on a one (not a reason) to seven (a very important reason) scale. Evidence for the internal consistency (Cronbach’s alphas range from 0.80 to 0.93), test-retest reliability (*r*s range from 0.71 to 0.90), and factorial and construct validity of the scales has been presented previously (Masters et al., 1993; Masters and Ogles, 1995; Ogles et al., 1995).

Marathoners were asked to respond to the MOMS items with respect to their running in general, rather than in specific relation to their most recent marathon (as was the case with RTP and WSM items). For brevity, we presented only 27 of the 56 MOMS items; from each of the nine sub-scales, we chose the three items that showed the highest factor loadings in Masters et al. (1993). The three competitiveness items were: “To see how high I can place in races”; “To get a faster time than my friends”; and (3) “To beat someone I’ve never beaten before.” From the 1,570 participants who completed all three competitiveness items, we found a Cronbach’s alpha of 0.758. The three goal achievement items were: “To improve my running speed”; “To try to run faster,” and “To see if I can beat a certain time.” From the 1,569 participants who completed all three of the goal achievement items, we found a Cronbach’s alpha of 0.857.

Finally, marathoners were also asked to complete items from the DOSPERT scale (Blais and Weber, 2006), which consists of 30 items addressing the likelihood of engaging in various risky activities. Much evidence supports the reliability and validity of the DOSPERT (Blais and Weber, 2006), and it is probably the most widely used self-report instrument for assessing risk taking (Highhouse et al., 2017). Participants rate each item on a 7-point Likert scale, ranging from extremely unlikely (1) to extremely likely (7). There are five items for each of six risk domains, namely social, recreational, health/safety, financial gambling, financial investment, and ethical. In order to reduce the time burden on participants, we abbreviated the 30 items to 12 items, which we summed to obtain the DOSPERT score. We abbreviated the scale by only using the first three items for each domain, rather than all five items. We also eliminated the items addressing the domains of ethical and financial investment. These domains seemed least likely to be related to risking taking in pace (RTP). The first item from the social domain was “Admitting that your tastes are different from those of a friend”; the first item from the recreational domain was “Going camping in the wilderness”; the first item from health/safety was “Drinking heavily at a social function”; the first item from financial gambling was “Betting a day’s income at the horse races.” From the 1,542 participants who completed all twelve of the DOSPERT items, we found a Cronbach’s alpha of 0.634.

### Definition of Pacing

In this study, we operationalized pacing as the percentage difference between the second half of the marathon completion time and first half of the marathon completion time [pacing = (second half time - first half time)/first half time ^∗^ 100] (Deaner et al., 2015a). Positive pacing values indicate that a runner ran slower in the second half of the marathon than they did in the first half. To make interpretations more intuitive, we generally describe cases with positive pacing values as slowing.

### Analysis

All analyses were performed using the R statistical computing language, version 3.4.0 (R Core Team, 2017).

When investigating the relationship between finishing time and pacing, we adjusted female finishing times by 12% to address the fact that female performances are roughly 10–12% slower than male performances even when training and talents are similar (Deaner et al., 2015a). The adjustment was made by dividing female finishing times by 1.12.

For ease of interpretability, in regression models and for the purpose of computing correlations, we treated the following training values as quantitative: typical training distance per week (in miles), number of weeks trained, number of preparatory long training runs per week, number of previously completed marathons, and years of training and/or competing in distance running, even though the survey respondents were presented with ordinal options for these items. Generally, we did this by replacing ordinal survey responses with their numerical midpoint. For example, respondents who said they trained “0–9” miles per week were assigned a value of 4.5. The highest ordinal response was replaced with the minimum of its range (e.g., 120+ miles per week was replaced with 120). We have presented data in miles rather than kilometers in this paper as this is the unit of distance most frequently used by runners in the United States and United Kingdom (where most participants came from), although all items addressing distance included kilometer equivalents. In figures, we further jittered (i.e., slightly offset) these values for readability. Treating these variables as quantitative made no substantive differences to our findings. Model results with these variables treated as categorical are available upon request from the authors.

**FIGURE 1:**
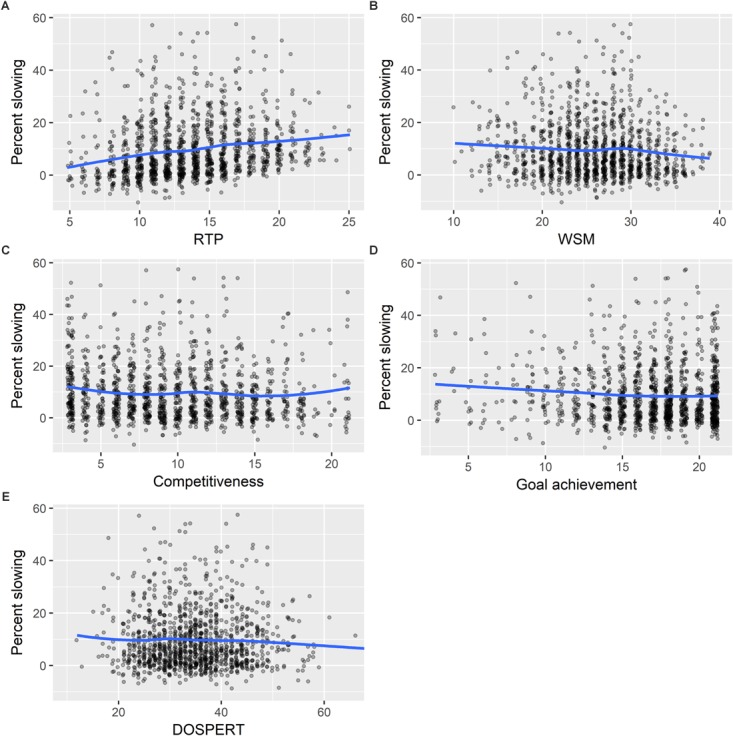
Visualizations of the relationships between pacing (measured by percent slowing in the second half of a marathon) and the five psychological constructs: **(A)** Risk taking in pace (RTP), **(B)** Willingness to suffer in the marathon (WSM), **(C)** Competitiveness, **(D)** Goal achievement, and **(E)** Domain-specific risk taking (DOSPERT).

**FIGURE 2:**
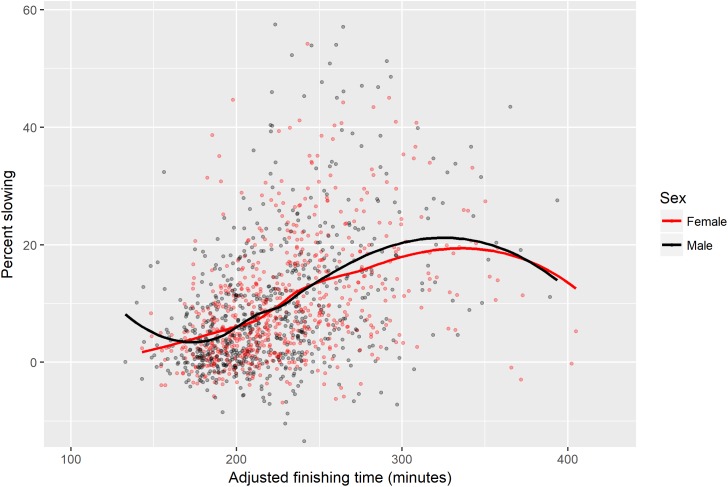
Scatter plot of percent slowing by adjusted finishing time, categorized by sex.

**FIGURE 3:**
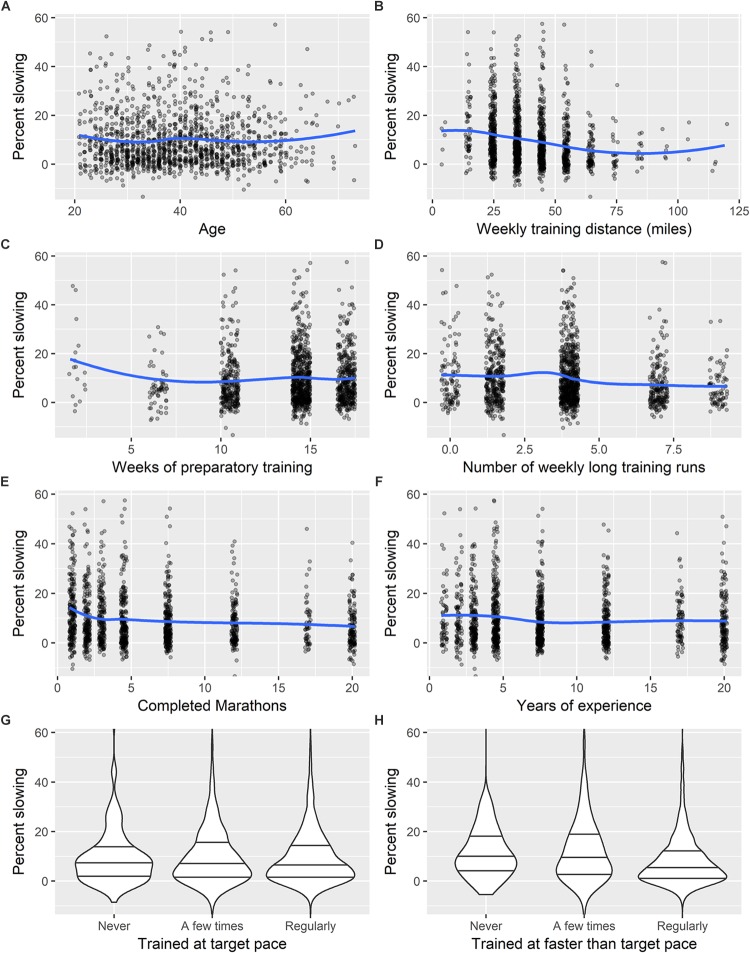
Visualizations of percent slowing by eight control variables presented in Table 4. **(A)** Age, **(B)** weekly training distance, **(C)** weeks of preparatory training, **(D)** number of weekly long training runs, **(E)** completed marathons, **(F)** years of experience, **(G)** trained at target pace, **(H)** trained at faster than target pace. In the violin plots, the horizontal lines show the 25th, 50th, and 75th percentile of slowing, respectively.

For all scatterplots presented in Figure 1–3, and in the Supplementary Material, we add a LOESS smoothed curve to the graph to help observe general patterns in the data.

## Results

We first explored the correlations among the five psychological constructs. As shown in Table 2, these correlations were generally modest, indicating that the constructs are largely independent.

**Table 2:** Correlation matrix for the five psychological constructs: Risk taking in pace (RTP), Willingness to suffer in the marathon (WSM), Competitiveness, Goal achievement, and Domain-specific risk taking (DOSPERT).

	RTP	WSM	Competitiveness	Goal achievement	DOSPERT
RTP	1	0.265	0.123	0.0858	0.111
WSM	0.265	1	0.249	0.328	0.196
Competitiveness	0.123	0.249	1	0.518	0.119
Goal achievement	0.0858	0.328	0.518	1	0.138
DOSPERT	0.111	0.196	0.119	0.138	1


We next examined how these psychological constructs related to pacing. Table 3 shows five univariate models, each examining how one of the constructs predicted pacing. This table also displays the results of the multiple regression model which employed all five psychological constructs simultaneously. We found that greater RTP was strongly correlated with greater pacing values (i.e., with greater slowing in the marathon). From the simple linear regression model, each 1 point increase in RTP was associated with an increase of 0.57% points in our pacing measure (i.e., the slowing percentage is 0.57 larger). The magnitude of the effect increased to 0.642% points when we controlled for the other four constructs.

**Table 3:** Regression results of pacing modeled by the five psychological constructs: Risk taking in pace (RTP), Willingness to suffer in the marathon (WSM), Competitiveness, Goal achievement, and Domain-specific risk taking (DOSPERT).

Construct	Simple linear regressions			Multiple regression		
		
	Coefficient	*SE*	*p*-value	Coefficient	*SE*	*p*-value
RTP	0.575	0.0856	2.8e-11	0.642	0.0920	5.2e-12
WSM	-0.140	0.0621	0.0245	-0.220	0.0699	1.7e-03
Competitiveness	-0.157	0.0729	0.0317	-0.0876	0.0870	0.314
Goal achievement	-0.199	0.0872	0.0227	-0.0871	0.108	0.418
DOSPERT	-0.0361	0.0405	0.373	-0.0423	0.0415	0.309
				*R*^2^ = 0.0472		


Willingness to suffer in the marathon, competitiveness, DOSPERT, and goal achievement were negatively associated with pacing; that is, greater WSM, greater competitiveness, greater DOSPERT, and greater goal achievement were each associated with lesser slowing. The statistical significance, however, was less pronounced for these constructs; in neither the univariate nor the multivariate model did DOSPERT attain significance at the 5% level. In each univariate model, WSM, competitiveness, and goal achievement were statistically significant at the 5% level, but not at the 1% level. In the multiple regression models, the magnitude of the effects diminished for competitiveness and goal achievement and was no longer significant. Conversely, the coefficient on WSM was amplified, and its association with pacing was strong after controlling for the other four constructs. Figure 1 displays scatter plot visualizations of percent slowing by each of the five psychological constructs.

In Table 4, we explore the possible relationship between pacing and several control variables, employed both individually, and simultaneously in a single multiple regression model. The 10 control variables we considered are: adjusted finishing time, sex, age, typical training distance per week, number of weeks trained, number of preparatory long training runs per week, number of previously completed marathons, years of training and/or competing in distance running, frequency of training at target pace, and frequency of training at faster than target pace. The simple linear regression models presented in Table 4 indicate that runners with greater weekly training distances, more long training runs, more completed marathons, more years of experience, and who regularly trained at faster than their target pace showed significantly less slowing in the second half of their marathon.

**Table 4:** Regression results of pacing modeled by 10 control variables.

Construct	Simple linear regressions			Multiple regression		
		
	Coefficient	*SE*	*p*-value	Coefficient	*SE*	*p*-value
Training distance	-0.146	0.0213	9.98e-12	0.0711	0.0258	0.00602
Weeks trained	0.0351	0.109	0.747	-0.0607	0.102	0.550
Long runs	-0.543	0.143	1.55e- 04	0.0506	0.148	0.732
Completed marathons	-0.281	0.0565	7.37e-07	-0.125	0.0665	0.0610
Years of experience	-0.137	0.0576	0.0178	0.138	0.0645	0.0325
Target pace (a few times)	0.390	1.419	0.784	-0.170	1.316	0.897
Target pace (regularly)	-0.366	1.372	0.790	-0.336	1.300	0.796
Fast pace (a few times)	0.410	1.363	0.763	1.878	1.294	0.147
Fast pace (regularly)	-3.594	1.288	0.00533	0.227	1.280	0.859
				*R*^2^ = 0.192		


The strongest predictor of slowing was the quality of the runner (as measured by their adjusted finishing time); on average, every additional 8.6 min of increase in the finishing time of their last marathon was associated with a 1% point increase in our pacing measure. The magnitude of the effect increased to 1% point of added slowing per 7.5 min of additional finishing time when we accounted for the other control variables.

Consistent with prior work (March et al., 2011; Trubee et al., 2014; Deaner et al., 2015a; Krawczyk and Wilamowski, 2017; Nikolaidis and Knechtle, 2017), older runners paced more evenly. In contrast to previous research (March et al., 2011; Santos-Lozano et al., 2014; Trubee et al., 2014; Deaner et al., 2015a; Hubble and Zhao, 2016; Krawczyk and Wilamowski, 2017; Smyth, 2018), we did not find robust evidence of greater male slowing. Males slowed slightly less than females overall (9.3% vs. 10.2% points), and, in a multiple regression, although males slowed more than females (0.22% points), this difference did not reach statistical significance (*p* = 0.726). Finally, the multiple regression in Table 4 indicates a few non-intuitive results, in particular, that more years of experience and greater training distances were associated with more slowing.

Figure 2 displays a scatter plot of percent slowing by runner quality, categorized by sex. This figure indicates that among faster runners in our sample, males and females slowed at very similar levels, while males exhibited more slowing among slower runners. Figure 3 shows visualizations of percent slowing by the other eight control variables presented in Table 4.

In Table 5, we re-investigate the relationship between each psychological construct and pacing, but now controlling for the other constructs, and the 10 control variables presented in Table 4. For two of the constructs (competitiveness and goal achievement), the direction of the relation with slowing has reversed, and all but one of the constructs (RTP) is now insignificantly associated with slowing at the 5% level. With regards to RTP, it maintains a very strong, positive, relation with our pacing measure: runners who reported greater RTP generally reported greater slowing. The practical magnitude of this effect is substantial: for example, all else equal, a runner at the 75th quantile of RTP (a score of 16) would be predicted to experience 3.64% points more slowing than one at the 25th quantile (a score of 11).

**Table 5:** Modeling pacing by psychological constructs, controlling for other constructs and 10 control variables listed in Table 4.

Construct	Coefficient	*SE*	*p*-value
RTP	0.728	0.0828	<2e-16
WSM	-0.0130	0.0650	0.841
Competitiveness	0.106	0.0799	0.187
Goal achievement	0.191	0.0999	0.0565
DOSPERT	-0.0703	0.0382	0.0664
	*R*^2^ = 0.256		


*Results for the control variables are omitted for brevity.*

## Discussion

The present study showed that RTP – the extent to which runners agreed that they began the marathon at a pace that was so fast that it would jeopardize their capacity to maintain this pace throughout the event – was a robust predictor of slowing in the marathon (Figure 1). Greater RTP predicted greater slowing in regression models controlling for other psychological constructs (Table 3) and in regression models controlling for these constructs as well as training measures, and other known pacing correlates (i.e., sex, age, finishing time; Table 5). Moreover, these analyses indicated a non-trivial effect size. In particular, we estimated that, all else equal, a runner at the 75th quantile of RTP would experience 3.64% points more slowing than one at the 25th quantile.

The present study supports recent work indicating that psychological factors may be important in explaining pacing variation among non-elite marathoners (Hubble and Zhao, 2016; Krawczyk and Wilamowski, 2017). In particular, previous studies found that marathoners who slow more exhibit a larger discrepancy between their pre-race self-forecasts and their actual performances, a pattern suggesting that overconfidence leads to greater slowing (Hubble and Zhao, 2016; Krawczyk and Wilamowski, 2017). Our results are consistent with this interpretation because both an overconfident runner and a risk taking one will begin at a pace that is fast relative to their ability; however, the risk taking runner, but not the overconfident one, is aware that selecting this pace puts them in jeopardy of slowing dramatically. Our results also complement studies of cycling and ultramarathoning, which found that athletes with greater perceptions of risk (across all situations, not merely athletics) are more likely to adopt relatively slow initial paces (Micklewright et al., 2015). Our results are consistent with this because an athlete that perceives that slowing dramatically is not risky (i.e., a perception which is a function of the likelihood and negative value of an outcome; Blais and Weber, 2006) will be more likely to risk beginning with a fast pace. However, it may also be that even when athletes have similar risk perceptions, some individuals will be more likely to make risky choices, perhaps because they hold a higher subjective value of the possible positive outcome of their decision (e.g., achieving an outstanding marathon performance). It is generally assumed that runners consider trade-offs (Smits et al., 2014; Markle et al., 2018) when selecting their paces (i.e., chance of outstanding performance vs. chance of slowing dramatically and experiencing great discomfort). Our results arguably constitute the most direct evidence yet of the recognition of such tradeoffs, at least among non-elite marathoners.

Although we found robust evidence that RTP predicts pacing, this was not true for four other candidate psychological constructs: competitiveness, goal achievement, and WSM, and DOSPERT (Figure 1). Competitiveness and goal achievement showed the predicted (i.e., negative) associations with pacing in simple linear regressions (Table 3), but these associations were far weaker than the association between RTP and pacing. Furthermore, these associations were not significant in analyses controlling for the other psychological constructs, training measures, and other known pacing correlates (Tables 3, 5). In hindsight, it seems reasonable that competitiveness and goal achievement would not be robustly associated with pacing given the tradeoffs between aggressive and cautious paces noted above. For example, a highly competitive, goal-oriented runner might prioritize achieving a good performance in every marathon and therefore might adopt a fairly cautious initial pace; another a highly competitive, goal-oriented runner might prioritize the possibility of achieving an exceptional performance even at the risk of slowing dramatically. Another point is that, although competitiveness and goal achievement assessed with the MOMs scales are reliably correlated with running performance (Masters et al., 1993; Ogles and Masters, 2003; Deaner et al., 2015b), these relations might be chiefly driven by competitive, goal-oriented runners maintaining unusually consistent, demanding training, rather than by such runners adopting exceptional pacing or racing strategies.

Willingness to suffer in the marathon is, like RTP, a construct introduced for the first time in the present study, and it was also predicted to show a strong relationship with pacing. In simple linear regressions and in models controlling for the other three psychological constructs (Table 3), greater WSM was associated with lesser slowing, which is the opposite of what we predicted. Moreover, WSM was not significantly associated with slowing in analyses controlling for the other psychological constructs, training measures, and other known pacing correlates (Table 5). In hindsight, the prediction that greater WSM would be associated with greater slowing was perhaps naive. We predicted this because we expected that runners would believe that beginning with a risky pace would frequently lead to suffering later in the race (e.g., cramping, “hitting the wall”); therefore, risk taking runners would generally be those who would be willing to suffer. This reasoning, however, ignores other possibilities. For instance, a runner with modest time goals (given their ability) might plan to begin a race at a comfortable, cautious pace and then plan to slow substantially later in the race to ensure they experienced little discomfort; this runner would report low WSM and substantial slowing. Another possibility is that a runner might plan to begin at a fairly aggressive pace and plan to maintain this pace because they are willing to suffer later in the race; this runner would report high WSM and, if successful, little slowing. Thus, although we still believe that WSM is likely to be related to pacing in some ways, it now seems sensible that there will be no robust overall association. The modest correlation between WSM and RTP (*r* = 0.27; Table 2) further suggests that WSM has a weaker association with pacing than we initially expected.

DOSPERT was not significantly related to pacing in univariate models (Table 3), in models that controlled for the other psychological constructs (Table 3), or in models that controlled for the other psychological constructs, training measures, and other known pacing correlates (Table 5). Moreover, DOSPERT was only weakly correlated with RTP (*r* = 0.11; Table 2). In other words, our results indicate that runners who are unusually prone to take risks in their marathon pacing are not especially likely to take risks in other situations. Thus, these results do not support the hypothesis of a domain-general risk taking propensity that generalizes across situations (Frey et al., 2017; Highhouse et al., 2017). These results are instead consistent with the view that risk taking is domain-specific (Blais and Weber, 2006; Figner and Weber, 2011; Fox and Tannenbaum, 2011).

Our analyses also revealed additional pacing correlates (Figure 3). Confirming prior research with non-elite marathoners (March et al., 2011; Renfree and Gibson, 2013; Santos-Lozano et al., 2014; Trubee et al., 2014; Deaner et al., 2015a; Hubble and Zhao, 2016; Krawczyk and Wilamowski, 2017; Nikolaidis and Knechtle, 2017; Smyth, 2018), finishing time and age were significant predictors of slowing in both simple linear regressions and multiple regressions controlling for other potential pacing correlates (Table 4). We also found that several training and experience variables were correlated with pacing in simple linear regressions (Table 4), and these associations support the recommendations of many coaches and training manuals. Specifically, lesser slowing was associated with greater training distance, more long runs, training at faster than marathon pace, more years of experience, and more completed marathons. Intriguingly, however, these associations did not hold in multiple regression models (Table 3). Perhaps most notably, after controlling for other potential pacing correlates, greater training distance was associated with significantly greater slowing. This result is difficult to interpret, but it might suggest, for example, that for an experienced runner who is regularly undertaking long runs (e.g., at least 29 kilometers every other week) during an extended marathon buildup (e.g., at least 16 weeks), a moderate training volume may be sufficient to run a well-paced marathon, and an unusually high training volume could be detrimental, perhaps because it does not allow sufficient recovery. Another possibility is that a runner who is exceptionally motivated to perform well might undertake a very large training volume (relative to their ability and experience) and might also be motivated to select a highly risky pace. This suggestion, however, is weakened by the fact that training distance and RTP were uncorrelated (*r* = 0.02).

### Limitations

This study had several limitations. First, we assessed pacing by assessing first and second half split times, so we were unable to identify when slowing typically began. Other studies report that slowing becomes pronounced in the final 10–15 km of the marathon (Buman et al., 2008; Ely et al., 2008; March et al., 2011). Second, we did not account for the substantial pacing variation that may be associated with particular weather conditions and marathon courses (Ely et al., 2008; Trubee et al., 2014). Third, our measures of training and experience, although showing some associations with pacing, were approximate. Fourth, our sample was self-selected, and, as detailed in the Section “Materials and Methods,” the participants differed in demographics, finishing time, and pacing compared to finishers of large United States marathons (Deaner et al., 2015a). One indicator of our sample’s non-representativeness is that we found no clear evidence of greater male slowing (Table 4 and Figure 2), a phenomenon which has emerged consistently in other studies of non-elite marathoners (March et al., 2011; Santos-Lozano et al., 2014; Trubee et al., 2014; Deaner et al., 2015a; Hubble and Zhao, 2016; Krawczyk and Wilamowski, 2017; Smyth, 2018).

A fifth limitation is that we introduced the constructs of RTP and WSM in this study, and the reliability and validity of our scales has not yet been established. Future studies might investigate the scales’ test-retest reliability and introduce new items or modify existing ones in order to increase the items’ internal consistency. Future studies might also test the validity of these scales, by testing, for instance, if they predict self-selected pacing in laboratory conditions (Micklewright et al., 2015) or if they show discriminant validity relative to related concepts, such as mental toughness (Gucciardi et al., 2009; Connaughton et al., 2010).

A sixth limitation of our study, and probably its most significant one, is its retrospective design. We asked runners to report their training, performance, psychology, and pacing in their most recently completed marathon, and runners’ recollections may be inaccurate or biased. For instance, runners were asked to name the most recent marathon where they intended to perform as well as they could. However, if a runner had intended to run well in their most recent marathon but actually performed very poorly, the runner may, in retrospect, decide that this marathon had been intended merely as a training run; they would thus provide information on their previous marathon where they had performed better. Such a bias might explain, at least partly, why our participants, especially the males, reported that they slowed less in their marathons and had faster overall finishing times than did finishers in 14 large United States marathons. Additionally, our design might have encouraged respondents to fabricate a *post hoc* explanation for their experiences and motivation. For example, a runner who slowed greatly might rationalize (and report) this as being due to an especially aggressive, risky racing plan, when in fact their racing plan had not had been unusual. Prospective studies, although highly difficult to undertake, would be invaluable in addressing these potential biases.

## Conclusion

This study’s main finding – that RTP was a robust predictor of marathon slowing – provides strong evidence that non-elite marathoners consider trade-offs when making pacing decisions. Future research should seek to corroborate this conclusion by replicating it using a prospective design (Hubble and Zhao, 2016; Krawczyk and Wilamowski, 2017) and addressing the present study’s other limitations. Future research could also explore a host of questions regarding marathoners’ pacing decisions, including runners’ assessments of their likelihood of maintaining various paces, the accuracy of such assessments, and runners’ post-race assessments of their pacing decisions. Research on this topic could yield performance insights for scientists, coaches, and athletes.

## Data Availability

The dataset generated and analyzed for this study can be found in the Supplementary Material.

## Author Contributions

RD, VA, and BH conceptualized and designed the study and wrote the manuscript. VA conducted the analyses and created the figures and tables. All authors read and approved the final manuscript.

## Conflict of Interest Statement

The authors declare that the research was conducted in the absence of any commercial or financial relationships that could be construed as a potential conflict of interest.
