# Supplementary material for: Risk Taking Runners Slow More in the Marathon
Source: Front Psychol. 2019 Feb 27;10:333. doi: 10.3389/fpsyg.2019.00333 (PMC6400853; doi:10.3389/fpsyg.2019.00333)
Supplement: Supplementary file 1 [file Table_1.DOCX]

Supplementary Material

Ambitious Runners Slow More in the Marathon

Robert O. Deaner*, Vittorio Addona, Brian Hanley

*** Correspondence:** Robert O. Deaner: deanerr@gvsu.edu

# Survery “Pacing in Marathon Runners” - version April 19 2016

Start of Block: Default Question Block

------------ INFORMED CONSENT - Pacing in Marathon Runners  ------------ PURPOSE OF THE STUDYThe purpose of this research study is to explore the relationships among experience, training,performance, motivation, and pacing in marathon runners. Only individuals who are at least 21 years of age and who have completed at least one marathon are eligible to participate. WHAT YOU WILL DOIf you choose to participate, you will be asked to answer brief questions about your experience as a distance runner. You will be asked questions about your training, performance, motivation, and pacing in your most recently completed marathon.There will also be questions addressing the reasons why you run and your decision making in non-running situations. ***Your total active involvement will be approximately 8 – 20 minutes.*** POTENTIAL BENEFITS AND RISKSIt is possible, although unlikely, that the issues raised by these survey items may cause emotional discomfort. If you feel uncomfortable while completing one or more of the questions, please skip these questions.  COMPENSATION FOR PARTICIPATIONYou will not be paid or otherwise compensated for your participation in this study. CONFIDENTIALITYWe will not ask for your name. Data generated by this study will not be linked to your name in reports or publications.  PARTICIPATION AND WITHDRAWALYour participation is voluntary. You may refuse to participate or you may withdraw from the study, for any reason, without penalty.   IDENTIFICATION OF INVESTIGATORSPrimary Investigator: Dr. Robert O Deaner Ph.D., Associate Professor, Dept. of Psychology, Grand Valley State University Allendale, MI; email: deanerr@gvsu.edu.Co-Investigator: Dr. Brian Hanley, Ph.D., Senior Lecturer in Sport and Exercise Biomechanics, Leeds Beckett University, Leeds, UK; email: B.Hanley@leedsbeckett.ac.uk. INSTITUTIONAL REVIEWThis study has been determined exempt by the Grand Valley State University Human Research Review Committee (870392-1; 16-120-H). If you have any questions about your rights as research participant, you may contact the Grand Valley State University Human Research Review Committee, 049 James Zumberge Hall Allendale, MI 4940, email: rpp@gvsu.edu. You can also directly contact the chair of the committee, Dr. Christine Yalda (616-331-7135) or the head of the Psychology department, Dr. Robert Hendersen (616-331-2195).

| 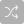 |
| --- |

Would you like to participate?

- Yes, I would like to participate. (1)
- No, I would not like to participate. (2)

Skip To: End of Survey If Would you like to participate?  = No, I would not like to participate.

| 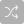 |
| --- |

Are you eligible to participate? To be eligible, you must be at least 21 years of age and must have completed at least one marathon.

- Yes, I am eligible. (1)
- No, I am not eligible. (2)

Skip To: End of Survey If Are you eligible to participate? To be eligible, you must be at least 21 years of age and must ha... = No, I am not eligible.

| Page Break |  |
| --- | --- |

| 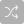 |
| --- |

What year were you born?

▼ 2000 (1) ... 1900 (101)

| 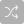 |
| --- |

What is your gender?

- Male (1)
- Female (2)

| 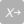 |
| --- |

In which country do you mainly reside?

▼ Afghanistan (1) ... Zimbabwe (193)

If you know it, what is your lifetime personal best time for the marathon (26.2 miles, 42.195 km)?

- Hours (1) ________________________________________________
- Minutes (2) ________________________________________________
- Seconds (3) ________________________________________________

| 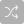 |
| --- |

When did you achieve this best marathon performance?

▼ in the past 12 months (1) ... 11 or more years ago (6)

If you know it, what is your lifetime personal best time for the half marathon (13.1 miles, 21.097 km)?

- Hours (1) ________________________________________________
- Minutes (2) ________________________________________________
- Seconds (3) ________________________________________________

| 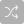 |
| --- |

When did you achieve this best half marathon performance?

▼ in the past 12 months (1) ... 11 or more years ago (6)

| 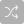 |
| --- |

How many total MARATHONS have you FINISHED in your lifetime?

▼ 1 (2) ... 20+ (9)

| 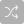 |
| --- |

How many total MARATHONS have you started but NOT finished in your lifetime?

▼ 0 (10) ... 20+ (9)

| 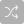 |
| --- |

How many YEARS have you been training and/or competing in distance running?

▼ 1 (2) ... 20+ (9)

If you suffer from any major health conditions (e.g., heart disease), please list them.

________________________________________________________________

________________________________________________________________

________________________________________________________________

________________________________________________________________

________________________________________________________________

| Page Break |  |
| --- | --- |

What was the MOST RECENT marathon you FINISHED (e.g. Boston, London) WHERE YOU INTENDED TO PERFORM AS WELL AS YOU COULD? For example, if you completed a marathon only as a training run or only to pace a friend and not to run as fast as possible, please disregard that marathon and consider the marathon you finished before that one.

________________________________________________________________

| 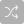 |
| --- |

In what year was this marathon held?

▼ 2016 (1) ... 1950 (67)

When answering the next several pages of the survey, PLEASE CONSIDER ONLY THIS MOST RECENT MARATHON YOU FINISHED (and where you intended to perform as well as you could).
 If you have a running journal, notes, splits (recorded by yourself or the official race timers) or any other previously recorded information from this marathon, PLEASE use this information when answering these questions.

Will you be using any previously recorded information to answer these questions?

- Yes (1)
- No (2)

| Page Break |  |
| --- | --- |

Did you ever deliberately run AT YOUR TARGET PACE in training as practice for this marathon?

- Regularly (1)
- A few times (2)
- Never (3)

Did you ever deliberately run substantially FASTER THAN YOUR TARGET PACE in training as practice for this marathon? This might be tempo training, threshold training, intervals, fartlek, 10K pace training, or any other training substantially faster than your target marathon pace

- Regularly (1)
- A few times (2)
- Never (3)

| 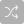 |
| --- |

What was your TYPICAL TRAINING DISTANCE covered each week during preparation for this marathon (not including any tapering period)? You can report either miles or kilometers.

▼ 0-9 miles per week (1) ... 200+ kilometers per week (66)

| 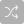 |
| --- |

About how many WEEKS did you train for this marathon, not including any tapering period?

▼ 4 weeks or fewer (1) ... 17 weeks or more (5)

| 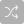 |
| --- |

About how many TRAINING RUNS or PREPARATION RACES did you do in preparation for this marathon that were at least 18 miles (29 kilometers) long?

▼ 0 runs/races of at least this length (1) ... 9 or more runs/races of at least this length (5)

What was your finishing time for this most recent marathon? If you know both a “gun time” and a “chip time,” please report the “chip time.”

- Hours (1) ________________________________________________
- Minutes (2) ________________________________________________
- Seconds (3) ________________________________________________

If, before you started this marathon, you had an EXPECTED (most likely) finishing time, please list it.

- Hours (1) ________________________________________________
- Minutes (2) ________________________________________________
- Seconds (3) ________________________________________________

If you listed an expected (most likely) finishing time, did you list this based on previously recorded information (e.g., journal, pacing plan) or only your current recollection?

- This expected time is based on previously recorded information. (1)
- This expected time is based only on my current recollection. (2)
- Not applicable – I didn’t list an expected finishing time. (3)

| Page Break |  |
| --- | --- |

If you know any split times for this most recent marathon, please record them below. 
We have listed possible splits in several ways because race websites report results for different splits (e.g., 10K, 5K, mile) and in different ways (e.g., sometimes for each segment and sometimes cumulatively for several segments).
Please don't feel obligated to report all possible splits or to make any new calculations. Simply record what is appropriate and easy for you.

First half of marathon

- Hours (1) ________________________________________________
- Minutes (2) ________________________________________________
- Seconds (3) ________________________________________________

***** Cumulative Splits for every 5K or every 10K *****(Please skip these if you are going to report 5K or 10K segment splits below)

Cumulative or total time at 5K

- Hours (1) ________________________________________________
- Minutes (2) ________________________________________________
- Seconds (3) ________________________________________________

Cumulative or total time at 10K

- Hours (1) ________________________________________________
- Minutes (2) ________________________________________________
- Seconds (3) ________________________________________________

Cumulative or total time at 15K

- Hours (1) ________________________________________________
- Minutes (2) ________________________________________________
- Seconds (3) ________________________________________________

Cumulative or total time at 20K

- Hours (1) ________________________________________________
- Minutes (2) ________________________________________________
- Seconds (3) ________________________________________________

Cumulative or total time at 25K

- Hours (1) ________________________________________________
- Minutes (2) ________________________________________________
- Seconds (3) ________________________________________________

Cumulative or total time at 30K

- Hours (1) ________________________________________________
- Minutes (2) ________________________________________________
- Seconds (3) ________________________________________________

Cumulative or total time at 35K

- Hours (1) ________________________________________________
- Minutes (2) ________________________________________________
- Seconds (3) ________________________________________________

Cumulative or total time at 40K

- Hours (1) ________________________________________________
- Minutes (2) ________________________________________________
- Seconds (3) ________________________________________________

***** 5K segment splits *****

First 5K

Minutes (1)

Seconds (2)

▼ 10 (1) ... 43+ ~ 0 (2015)

Second 5K (6-10 km)

Minutes (1)

Seconds (2)

▼ 10 (1) ... 43+ ~ 0 (2015)

Third 5K (11-15 km)

Minutes (1)

Seconds (2)

▼ 10 (1) ... 43+ ~ 0 (2015)

Fourth 5K (16-20 km)

Minutes (1)

Seconds (2)

▼ 10 (1) ... 43+ ~ 0 (2015)

Fifth 5K (21-25 km)

Minutes (1)

Seconds (2)

▼ 10 (1) ... 43+ ~ 0 (2015)

Sixth 5K (26-30 km)

Minutes (1)

Seconds (2)

▼ 10 (1) ... 43+ ~ 0 (2015)

Seventh 5K (31-35 km)

Minutes (1)

Seconds (2)

▼ 10 (1) ... 43+ ~ 0 (2015)

Eighth 5K (36-40 km)

Minutes (1)

Seconds (2)

▼ 10 (1) ... 43+ ~ 0 (2015)

Final 2.2 km of marathon

Minutes (1)

Seconds (2)

▼ 1 (1) ... 34+ ~ 0 (2015)

***** 10K segment splits *****(Please skip these if you have already reported 5K segment splits above)

First 10K

- Hours (1) ________________________________________________
- Minutes (2) ________________________________________________
- Seconds (3) ________________________________________________

Second 10K (11-20 km)

- Hours (1) ________________________________________________
- Minutes (2) ________________________________________________
- Seconds (3) ________________________________________________

Third 10K (21-30 km)

- Hours (1) ________________________________________________
- Minutes (2) ________________________________________________
- Seconds (3) ________________________________________________

Fourth 10K (31-40 km)

- Hours (1) ________________________________________________
- Minutes (2) ________________________________________________
- Seconds (3) ________________________________________________

Final 2.2 km of marathon

Minutes (1)

Seconds (2)

▼ 1 (1) ... 34+ ~ 0 (2015)

***** Mile splits *****

Mile 1

Minutes (1)

Seconds (2)

▼ 1 (1) ... 34+ ~ 0 (2015)

Mile 2

Minutes (1)

Seconds (2)

▼ 1 (1) ... 34+ ~ 0 (2015)

Mile 3

Minutes (1)

Seconds (2)

▼ 1 (1) ... 34+ ~ 0 (2015)

Mile 4

Minutes (1)

Seconds (2)

▼ 1 (1) ... 34+ ~ 0 (2015)

Mile 5

Minutes (1)

Seconds (2)

▼ 1 (1) ... 34+ ~ 0 (2015)

Mile 6

Minutes (1)

Seconds (2)

▼ 1 (1) ... 34+ ~ 0 (2015)

Mile 7

Minutes (1)

Seconds (2)

▼ 1 (1) ... 34+ ~ 0 (2015)

Mile 8

Minutes (1)

Seconds (2)

▼ 1 (1) ... 34+ ~ 0 (2015)

Mile 9

Minutes (1)

Seconds (2)

▼ 1 (1) ... 34+ ~ 0 (2015)

Mile 10

Minutes (1)

Seconds (2)

▼ 1 (1) ... 34+ ~ 0 (2015)

Mile 11

Minutes (1)

Seconds (2)

▼ 1 (1) ... 34+ ~ 0 (2015)

Mile 12

Minutes (1)

Seconds (2)

▼ 1 (1) ... 34+ ~ 0 (2015)

Mile 13

Minutes (1)

Seconds (2)

▼ 1 (1) ... 34+ ~ 0 (2015)

Mile 14

Minutes (1)

Seconds (2)

▼ 1 (1) ... 34+ ~ 0 (2015)

Mile 15

Minutes (1)

Seconds (2)

▼ 1 (1) ... 34+ ~ 0 (2015)

Mile 16

Minutes (1)

Seconds (2)

▼ 1 (1) ... 34+ ~ 0 (2015)

Mile 17

Minutes (1)

Seconds (2)

▼ 1 (1) ... 34+ ~ 0 (2015)

Mile 18

Minutes (1)

Seconds (2)

▼ 1 (1) ... 34+ ~ 0 (2015)

Mile 19

Minutes (1)

Seconds (2)

▼ 1 (1) ... 34+ ~ 0 (2015)

Mile 20

Minutes (1)

Seconds (2)

▼ 1 (1) ... 34+ ~ 0 (2015)

Mile 21

Minutes (1)

Seconds (2)

▼ 1 (1) ... 34+ ~ 0 (2015)

Mile 22

Minutes (1)

Seconds (2)

▼ 1 (1) ... 34+ ~ 0 (2015)

Mile 23

Minutes (1)

Seconds (2)

▼ 1 (1) ... 34+ ~ 0 (2015)

Mile 24

Minutes (1)

Seconds (2)

▼ 1 (1) ... 34+ ~ 0 (2015)

Mile 25

Minutes (1)

Seconds (2)

▼ 1 (1) ... 34+ ~ 0 (2015)

Mile 26

Minutes (1)

Seconds (2)

▼ 1 (1) ... 34+ ~ 0 (2015)

Final 0.2 miles

Minutes (1)

Seconds (2)

▼ 1 (1) ... 34+ ~ 0 (2015)

| Page Break |  |
| --- | --- |

What best describes your pace in the second half of this marathon in relation to your pre-race expectations?

- I ran the second half much faster than I expected. (1)
- I ran the second half somewhat faster than I expected. (2)
- I ran the second half about the same pace as I expected. (3)
- I ran the second half somewhat slower than I expected. (4)
- I ran the second half much slower than I expected. (5)

| 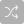 |
| --- |

To what extent were the following things reasons why you slowed your pace in the second half of this marathon?
(If you didn’t slow much in the second half, then it could be that none of these were reasons for your slowing.)

|  | 1 (Not a reason) (1) | 2 (2) | 3 (3) | 4 (4) | 5 (A very important reason) (5) |
| --- | --- | --- | --- | --- | --- |
| My pacing goals were unrealistic. (1) |  |  |  |  |  |
| I started more quickly than I intended to. (2) |  |  |  |  |  |
| I mistakenly followed the fast pace set by others. (3) |  |  |  |  |  |
| I was unable to locate or stay with the organized pacing group I planned to run with. (4) |  |  |  |  |  |
| I was carrying an injury or illness (already known before starting). (5) |  |  |  |  |  |
| I experienced a new injury or illness during the race. (6) |  |  |  |  |  |
| I had stomach cramps. (7) |  |  |  |  |  |
| I had leg cramps. (8) |  |  |  |  |  |
| I was too cold. (9) |  |  |  |  |  |
| The weather (e.g., wind, rain) presented problems. (10) |  |  |  |  |  |
| I became too hot. (11) |  |  |  |  |  |
| I became dehydrated. (12) |  |  |  |  |  |
| I wasn’t able to get enough nutrition (e.g., carbohydrates, electrolytes). (13) |  |  |  |  |  |
| I “hit the wall.” (14) |  |  |  |  |  |
| I was delayed at the toilet facilities. (15) |  |  |  |  |  |
| I had technology problems (e.g., watch stopped working; splits on course were incorrect). (16) |  |  |  |  |  |
| There were no other runners nearby to push me. (17) |  |  |  |  |  |
| There was little crowd support. (18) |  |  |  |  |  |
| I hadn’t trained well enough for this part of the race. (19) |  |  |  |  |  |
| I lost concentration or got distracted. (20) |  |  |  |  |  |
| I didn't taper properly. (21) |  |  |  |  |  |

| 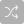 |
| --- |

Please indicate the extent to which you agree with the following statements ABOUT your pre-race pacing plan.

|  | Strongly disagree (1) | Disagree (2) | Neither agree or nor disagree (3) | Agree (4) | Strongly agree (5) |
| --- | --- | --- | --- | --- | --- |
| I had a plan for pacing that was based on how fast I would run different parts of the race. (1) |  |  |  |  |  |
| I had a plan for pacing that was based on how much I would exert myself during different parts of the race. (2) |  |  |  |  |  |
| I had a pacing plan to get under a specific time barrier (e.g., 2:50:00, 3:30:00, 4:00:00). (7) |  |  |  |  |  |
| I planned to run with an organized pace group. (3) |  |  |  |  |  |
| I planned to keep up with specific friends. (4) |  |  |  |  |  |
| I planned to keep up with specific competitors. (5) |  |  |  |  |  |
| I didn’t have a pre-race pacing plan. (6) |  |  |  |  |  |

| 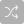 |
| --- |

Please indicate the extent to which you agree with the following statements about what contributed to your MAKING a pre-race pacing plan. (If you didn’t have a pre-race pacing plan, then it could be that none of these things mattered.)

|  | Strongly disagree (1) | Disagree (2) | Neither agree or nor disagree (3) | Agree (4) | Strongly agree (5) |
| --- | --- | --- | --- | --- | --- |
| I made my pacing plan based on my previous performance(s) in the marathon. (1) |  |  |  |  |  |
| I made my pacing plan based on my previous performance(s) at other distances. (2) |  |  |  |  |  |
| I made my pacing plan based on my recent training. (3) |  |  |  |  |  |
| I made my pacing plan using an online predictor or a running handbook or manual. (4) |  |  |  |  |  |
| I made my pacing plan based on the advice of a coach(s). (5) |  |  |  |  |  |
| I made my pacing plan based on the advice of a friend(s). (6) |  |  |  |  |  |

| Page Break |  |
| --- | --- |

| 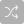 |
| --- |

Please indicate the extent to which you agree with the following statements about your pacing in this marathon.

|  | Strongly disagree (1) | Disagree (2) | Neither agree or nor disagree (3) | Agree (4) | Strongly Agree (5) |
| --- | --- | --- | --- | --- | --- |
| For me, achieving a satisfying performance meant running fairly aggressively early in the race. (1) |  |  |  |  |  |
| I was willing to be pretty conservative with my pace at the beginning so that I could be nearly certain that I’d finish strong (2) |  |  |  |  |  |
| I’d hate to finish and realize that I could have gotten a faster time if I had begun the race at a faster pace. (3) |  |  |  |  |  |
| I started at a pace that was slow enough that I expected to speed up considerably in the second half of the race. (4) |  |  |  |  |  |
| I realized that my starting pace was fast enough that I might not be able to maintain it. (5) |  |  |  |  |  |

| 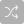 |
| --- |

Please rate each of the following items in terms of how important it was as a goal in this marathon. 
A score of 1 would indicate that the item was "not a goal" for this marathon. A score of 5 indicates that the item was "a very important goal" for this marathon. Intermediate scores represent relative degrees of each reason.

|  | 1 (not a goal) (1) | 2 (2) | 3 (3) | 4 (4) | 5 (a most important goal (5) |
| --- | --- | --- | --- | --- | --- |
| Discovering how much discomfort I could endure (1) |  |  |  |  |  |
| Making sure I finished feeling good (2) |  |  |  |  |  |
| Pushing myself to my limits (3) |  |  |  |  |  |
| Feeling comfortable during the race (4) |  |  |  |  |  |
| Staying healthy (5) |  |  |  |  |  |
| Testing my mental toughness (6) |  |  |  |  |  |
| Doing everything I could to achieve my fastest possible time (7) |  |  |  |  |  |
| Avoiding physical discomfort (8) |  |  |  |  |  |

Is there anything else you would like to tell us about your pacing that was not addressed in the previous questions? If so, please tell us here:

________________________________________________________________

________________________________________________________________

________________________________________________________________

________________________________________________________________

________________________________________________________________

| Page Break |  |
| --- | --- |

You have now finished the questions specifically addressing your most recently completed marathon. Please answer the next set of questions by thinking about YOUR RUNNING IN GENERAL.

| 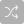 |
| --- |

Please rate each of the following items in terms of how important it is as a reason for why you run. A score of 1 would indicate that the item is "not a reason" for running. A score of 7 indicates that the item is "a very important reason" for running. Intermediate scores represent relative degrees of each reason.

|  | 1 (not a reason) (1) | 2 (2) | 3 (3) | 4 (4) | 5 (5) | 6 (6) | 7 (a very important reason) (7) |
| --- | --- | --- | --- | --- | --- | --- | --- |
| To help control my weight. (1) |  |  |  |  |  |  |  |
| To improve my running speed. (3) |  |  |  |  |  |  |  |
| To socialize with other runners. (5) |  |  |  |  |  |  |  |
| To improve my self-esteem. (6) |  |  |  |  |  |  |  |
| To prolong my life. (8) |  |  |  |  |  |  |  |
| To meet people. (9) |  |  |  |  |  |  |  |
| To make my life more purposeful. (10) |  |  |  |  |  |  |  |
| To look leaner. (11) |  |  |  |  |  |  |  |
| To try to run faster. (7) |  |  |  |  |  |  |  |
| To feel more confident about myself. (12) |  |  |  |  |  |  |  |
| To reduce my chance of having a heart attack. (13) |  |  |  |  |  |  |  |
| To make my life more complete. (14) |  |  |  |  |  |  |  |
| To improve my sense of self-worth. (15) |  |  |  |  |  |  |  |
| To share a group identity with other runners. (16) |  |  |  |  |  |  |  |
| To have time alone to sort things out. (4) |  |  |  |  |  |  |  |
| To concentrate on my thoughts. (2) |  |  |  |  |  |  |  |
| To solve problems. (17) |  |  |  |  |  |  |  |
| To see how high I can place in races. (18) |  |  |  |  |  |  |  |
| To get a faster time than my friends. (19) |  |  |  |  |  |  |  |
| To prevent illness. (20) |  |  |  |  |  |  |  |
| People look up to me. (21) |  |  |  |  |  |  |  |
| To see if I can beat a certain time. (22) |  |  |  |  |  |  |  |
| Brings me recognition. (23) |  |  |  |  |  |  |  |
| To beat someone I've never beaten before. (24) |  |  |  |  |  |  |  |
| To get compliments from others. (25) |  |  |  |  |  |  |  |
| To feel at peace with the world. (26) |  |  |  |  |  |  |  |
| To reduce my weight. (27) |  |  |  |  |  |  |  |

| Page Break |  |
| --- | --- |

You have now finished the questions addressing your running. Please answer the next set of questions by thinking about YOUR BEHAVIOR IN GENERAL.

| 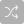 |
| --- |

For each of the following statements, please indicate the likelihood that you would engage in the described activity or behavior if you were to find yourself in that situation. Please provide a rating ranging from 1 (Extremely Unlikely) to 7 (Extremely Likely).

|  | 1 (extremely unlikely) (1) | 2 (2) | 3 (3) | 4 (4) | 5 (5) | 6 (6) | 7 (extremely likely) (7) |
| --- | --- | --- | --- | --- | --- | --- | --- |
| Admitting that your tastes are different from those of a friend. (1) |  |  |  |  |  |  |  |
| Going camping in the wilderness. (2) |  |  |  |  |  |  |  |
| Betting a day’s income at the horse races. (3) |  |  |  |  |  |  |  |
| Drinking heavily at a social function. (5) |  |  |  |  |  |  |  |
| Disagreeing with an authority figure on a major issue. (7) |  |  |  |  |  |  |  |
| Betting a day’s income at a high-stake poker game. (8) |  |  |  |  |  |  |  |
| Going down a ski run that is beyond your ability. (11) |  |  |  |  |  |  |  |
| Going whitewater rafting at high water in the spring. (13) |  |  |  |  |  |  |  |
| Betting a day’s income on the outcome of a sporting event. (14) |  |  |  |  |  |  |  |
| Engaging in unprotected sex. (15) |  |  |  |  |  |  |  |
| Driving a car without wearing a seatbelt (12) |  |  |  |  |  |  |  |
| Choosing a career that you truly enjoy over a more secure one. (4) |  |  |  |  |  |  |  |

| Page Break |  |
| --- | --- |

Have you taken this survey previously?

- Yes (1)
- No (2)

To what extent did you take the survey seriously?

- I answered everything honestly. (1)
- I was mostly honest. (2)
- I made up a lot of stuff. (3)

| Page Break |  |
| --- | --- |

You have finished the study.   Thank you for participating!

Because this survey is anonymous, we cannot automatically contact you to share the results. If you are interested in the results, please email deanerr@gvsu.edu and we will send you our paper when it is published.

If you have any questions or comments, please contact one of the investigators.   Primary Investigator: Dr. Robert O. Deaner, Ph.D., Associate Professor, Dept. of Psychology, Grand Valley State University, Allendale, MI; email: deanerr@gvsu.edu.
 Co-Investigator: Dr. Brian Hanley, Ph.D., Senior Lecturer in Sport and Exercise Biomechanics, Leeds Beckett University, Leeds, UK; email: B.Hanley@leedsbeckett.ac.uk.

End of Block: Default Question Block
