# Supplementary material for: Risk Taking Runners Slow More in the Marathon
Source: Front Psychol. 2019 Feb 27;10:333. doi: 10.3389/fpsyg.2019.00333 (PMC6400853; doi:10.3389/fpsyg.2019.00333)
Supplement: Supplementary file 3 [file Table_3.docx]

Supplementary Material

Risk Taking Runners Slow More in the Marathon

Robert O. Deaner*, Vittorio Addona, Brian Hanley

*** Correspondence:** Robert O. Deaner: deanerr@gvsu.edu

## Supplementary Tables and Figures

|  | RTP | WSM | Competitiveness | Goal achievement | DOSPERT |
| --- | --- | --- | --- | --- | --- |
| RTP | 1 | 0.239 | 0.075 | 0.023 | 0.0949 |
| WSM | 0.239 | 1 | 0.195 | 0.216 | 0.171 |
| Competitiveness | 0.075 | 0.195 | 1 | 0.518 | 0.119 |
| Goal achievement | 0.023 | 0.216 | 0.518 | 1 | 0.138 |
| DOSPERT | 0.0949 | 0.171 | 0.119 | 0.138 | 1 |

**Table 1s:** Correlation matrix for the psychological constructs: Risk taking in pace (RTP), Willingness to suffer in the marathon (WSM), Competitiveness, Goal achievement, and Domain-specific risk taking (DOSPERT). Altered version of Table 2 from main text, with RTP item 3, and WSM items 1 and 6, removed.

| Construct | Simple Linear Regressions | | | Multiple Regression | | |
| --- | --- | --- | --- | --- | --- | --- |
|  | Coefficient | SE | p-value | Coefficient | SE | p-value |
| RTP | 0.691 | 0.0971 | 1.93e-12 | 0.743 | 0.104 | 1.8e-12 |
| WSM | -0.230 | 0.0954 | 0.016 | -0.364 | 0.104 | 5.0e-04 |
| Competitiveness | -0.157 | 0.0729 | 0.0317 | -0.0701 | 0.0866 | 0.418 |
| Goal Achievement | -0.199 | 0.0872 | 0.0227 | -0.0888 | 0.105 | 0.400 |
| DOSPERT | -0.0361 | 0.0405 | 0.373 | -0.0388 | 0.0413 | 0.348 |
|  |  |  |  | R-squared = 0.0494 | | |

**Table 2s:** Regression results of pacing modeled by the five psychological constructs: Risk taking in pace (RTP), Willingness to suffer in the marathon (WSM), Competitiveness, Goal achievement, and Domain-specific risk taking (DOSPERT). Altered version of Table 3 from main text, with RTP item 3, and WSM items 1 and 6, removed.


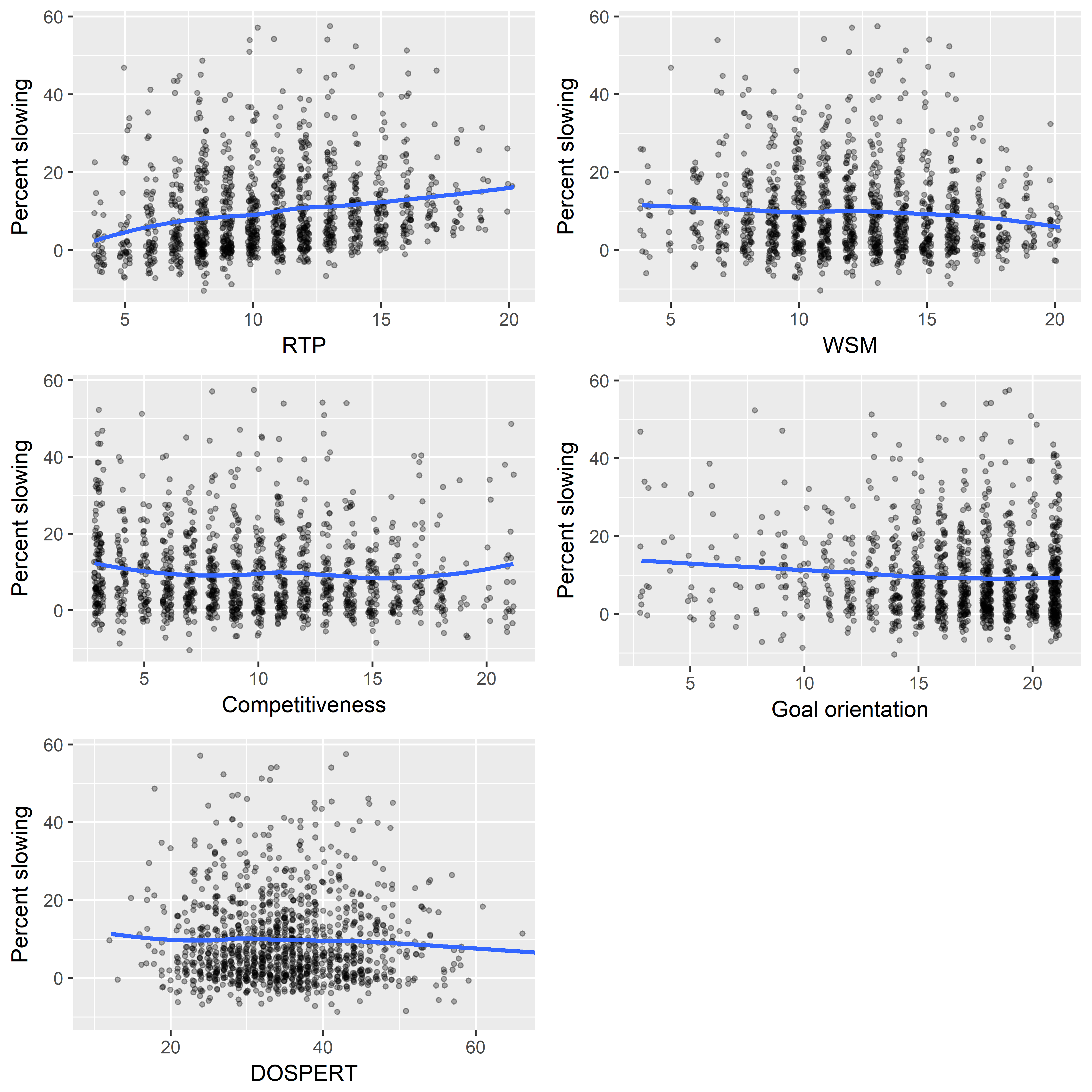


**Figure 1s:** Visualizations of the relationships between pacing (measured by percent slowing in the second half of a marathon) and the five psychological constructs: Risk taking in pace (RTP), Willingness to suffer in the marathon (WSM), Competitiveness, Goal achievement, and Domain-specific risk taking (DOSPERT). Altered version of Figure 1 from main text, with RTP item 3, and WSM items 1 and 6, removed.

| Construct | Coefficient | SE | p-value |
| --- | --- | --- | --- |
| RTP | 0.838 | 0.0939 | < 2e-16 |
| WSM | 0.0276 | 0.0989 | 0.780 |
| Competitiveness | 0.124 | 0.0794 | 0.120 |
| Goal achievement | 0.210 | 0.0984 | 0.0329 |
| DOSPERT | -0.0704 | 0.0380 | 0.0639 |
|  | R-squared = 0.259 | | |

**Table 3s:** Modeling pacing by psychological constructs, controlling for other constructs and 10 control variables listed in Table 4 (results for the control variables are omitted for brevity). Altered version of Table 5 from main text, with RTP item 3, and WSM to suffer items 1 and 6, removed.
